# Minimally Invasive Diastema Restoration with Prefabricated Sectional Veneers

**DOI:** 10.3390/dj8020060

**Published:** 2020-06-24

**Authors:** Claudio Novelli, Andrea Scribante

**Affiliations:** 1 Private Practice, DENS Centro Medico Lombardo, 20124 Milan, Italy; clnovelli@libero.it; 2 Unit of Orthodontics and Pediatric Dentistry, Section of Dentistry, Department of Clinical, Surgical, Diagnostic and Pediatric Sciences, University of Pavia, 27100 Pavia, Italy

**Keywords:** diastema, restoration, sectional veneer, ceramic veneer, composite veneer, prefabricated veneer

## Abstract

This case report presents a new technique for sectional veneer fabrication and diastema restoration with a prefabricated composite veneer. For the purpose of diastema restoration, a prefabricated sectional veneer provides the same benefits of a traditional ceramic sectional veneer (highly esthetic restoration with no need for tooth preparation) but involves a less technically demanding and time-consuming clinical procedure and a less delicate restoration with a reduced risk of accidental breakage and post-bonding crack formation. The technique presented in this case report bridges the gap between a direct and indirect technique for diastema restoration and introduces a new treatment option to close anterior spacing with a highly esthetic sectional veneer in a predictable and timely manner.

## 1. Introduction

Thanks to the combination of excellent esthetic outcomes and no sacrifices related to healthy tooth structure, orthodontic treatment is frequently advocated to close anterior diastemas [1,2,3]. However, when a Bolton discrepancy is detected, orthodontic treatment alone cannot establish proximal contacts with proper vertical and horizontal overlaps and restorative treatment is indicated to close the residual spaces [4,5,6]. Several treatment options are available for minimally invasive diastema restoration, including the recently introduced injectable composite resin technique, composite template technique and the prefabricated veneer technique [7,8,9,10]. However, direct composite restoration and indirect ceramic veneers are still the most popular minimally invasive treatment options to close the anterior spacing and restore the natural appearance of the smile. Direct composites can produce a very lifelike diastema restoration in a convenient single appointment with no need for tooth preparation. However, the technique requires advanced sculpting and finishing skills to achieve an optimally polished restoration with ideal anatomic form and no residual black triangle in the gingival embrasure [11,12,13]. Because of these limitations, some dentists prefer to depend on laboratory-fabricated ceramic veneers, especially for large diastemas that are more challenging to close with a direct technique. Ceramic veneers can produce a diastema restoration with exquisite esthetics and excellent longevity, but the technique requires a technically demanding and time-consuming clinical procedure, not to mention the sacrifice of a healthy tooth structure to allow space for the restorative material. Sometimes, a noninvasive veneer preparation is possible where the buccal surface remains intact, but the margin of the veneer still requires tooth reduction, especially in the interproximal area where the margin has to be moved lingually and sub-gingivally in order to produce effective space management and soft tissue contouring [14,15]. Ultimately, either direct composites or indirect ceramic veneers can serve very well for the purpose of diastema restoration; however, both techniques have intrinsic limitations for dentists who are looking for a minimally invasive and simple technique to close anterior spacings. This paper presents an alternative technique for diastema restoration with a prefabricated composite sectional veneer.

Prefabricated composite veneers are pre-shaped, pre-polished composite laminates available in different shapes and sizes for direct bonding to the deserving tooth with a complementary shade-matched composite resin [16,17,18]. This technique is indicated to treat a large variety of esthetic problems and to produce a veneer with a proper size, accurate anatomy and highly polished surface in a single appointment with a reduced number of clinical steps. One of the main innovations introduced by the prefabricated composite veneer technique is the possibility to customize the veneer chairside using conventional composite instruments. In the case presented in this report, the prefabricated composite veneer was customized chairside with a diamond separating disc to fit the size of the diastema to be closed. Then, a sectional veneer is ready for bonding to the spaced dentition and closes the diastema in a single appointment with no sacrifice of healthy tooth structure.

This technique bridges the gap between a direct and an indirect technique for diastema restoration and introduces a new treatment option to close anterior spacing with a highly esthetic sectional veneer in a predictable and timely manner.

## 2. Case Presentation

**Figure 1:**
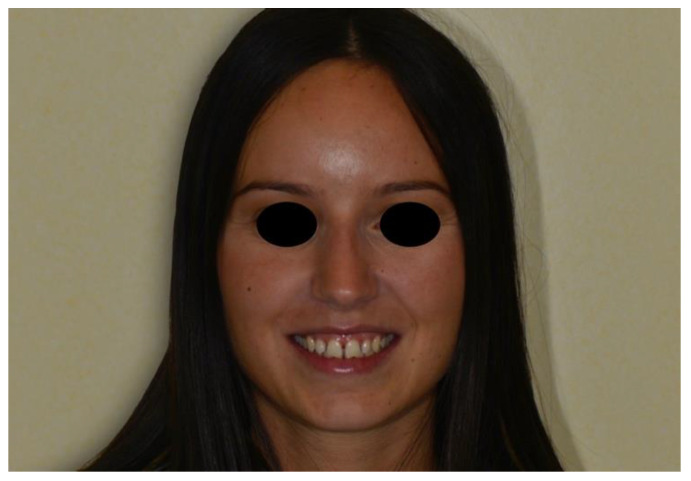
Preoperative full-face view.

**Figure 2:**
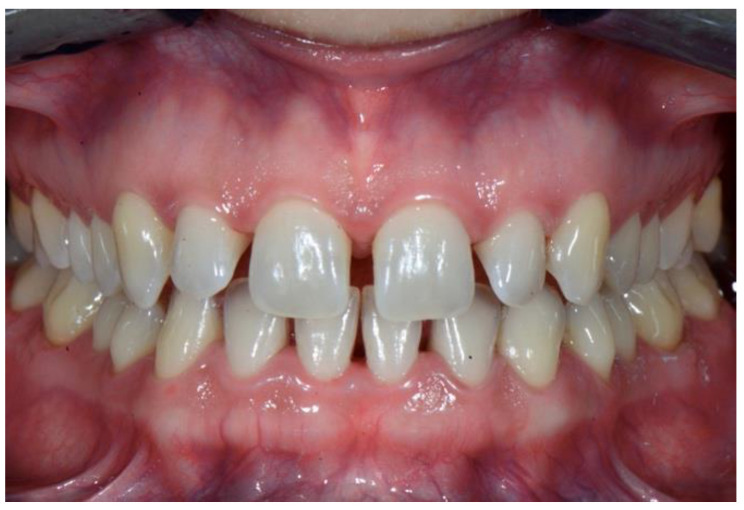
Preoperative retracted view.

The patient was a 27-year-old lady referred by her orthodontist for restorative treatment of multiple anterior maxillary diastemas (Figure 1). The unit internal review board approved the case report (2019-0923) on 23 September 2019. Clinical and radiological examination showed healthy incisors with no previous restorations and no signs of periodontal disease (Figure 2). Measurements from the study models revealed a significant Bolton discrepancy and a 1.9 mm diastema between central incisors associated with 1.1 mm bilateral diastemas between central and lateral incisors.

**Figure 3:**
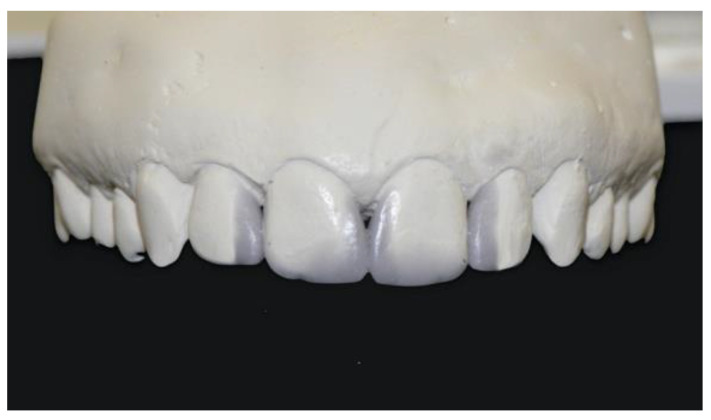
The diagnostic wax-up reveals a favorable 82% width-to-length ratio of the teeth restored with additional restorations.

Because of the young age of the patient, a noninvasive treatment with sectional veneers was recommended to close the spaces and restore the natural appearance of the smile. However, the size of the spaces to be closed was a possible contraindication for restoration with sectional veneers because the increased tooth width could result in altered width-to-length ratio and less than ideal tooth proportions. Ideal tooth proportions have been extensively discussed in the literature and many different ratios have been introduced to be used as esthetic guidelines for tooth dimensions [19,20,21]. However, these ratios rarely occur in smiles deemed attractive by laypeople and dental professionals and their rigid application to predict ideal tooth size is controversial [22,23,24,25,26]. An alternative approach to provide a guideline for ideal tooth dimensions is to use average tooth size in the human population as a reference. Anatomical values of tooth dimensions are well established in the literature and show that, although tooth width and length varies with gender, age and race, tooth width/length ratio in the upper anterior dentition is a stable, reference falling within a range of 72% to 85% [27,28,29]. For the patient presented in this report, the diagnostic wax-up revealed a favorable 82% width-to-length ratio and confirmed that, despite the moderate size of the diastemas, restoration with sectional veneers could be completed without unbalancing natural tooth proportions (Figure 3).

Once the preliminary measurements were completed and the treatment plan with sectional veneers was approved by the patient, the clinical procedure started with fitting the prefabricated veneers to the size of the diastema.

**Figure 4:**
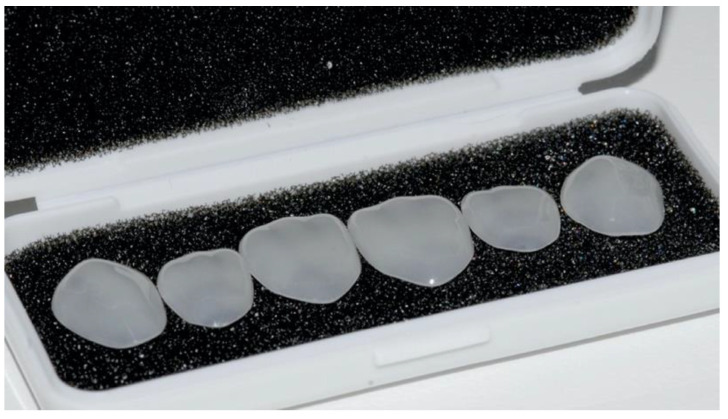
Prefabricated laser-sintered veneers.

The prefabricated composite veneers for the patient presented in this case report (Edelweiss Veneers, Edelweiss Dentistry, Wolfurt, Austria) are fabricated with a nanohybrid composite cured with high temperature and high pressure to produce a veneer with improved mechanical properties compared to a conventional composite restoration [30,31]. In addition to the heat/pressure curing process, the tested veneers also feature a proprietary laser sintering technology that extracts the resin component from the composite shell and produces a veneer with high inorganic content and a highly esthetic, laser-sintered surface [32,33] (Figure 4).

Edelweiss Prefabricated Veneers are available in four sizes (XS, S, M, L) and a custom sizing guide is included in the system to select the veneer that best fits the deserving tooth. For the patient in this case report, small veneers were selected.

**Figure 5:**
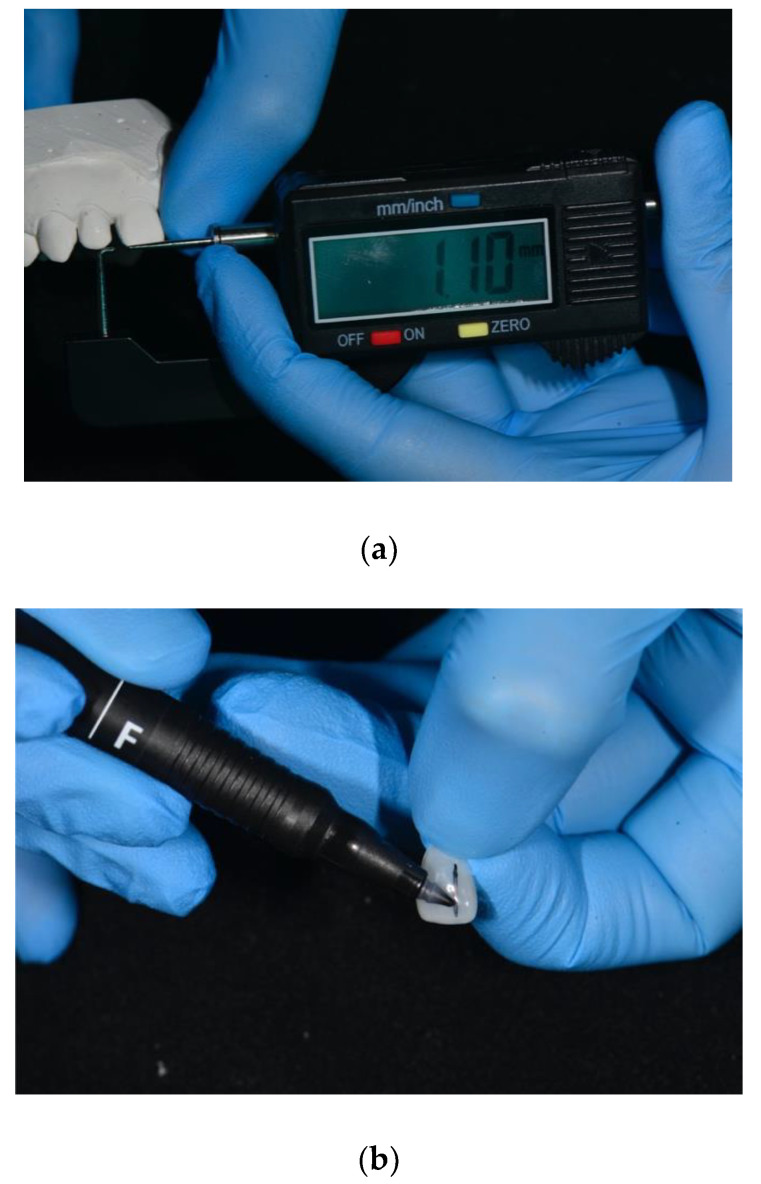
(**a**) The size of the diastema is measured with a digital caliper; (**b**) the size of the diastema is transferred to the veneer with a thin marker.

After veneer size selection, the width of the diastema was measured with a digital caliper and transferred to the veneer using a thin marker (Figure 5a,b).

**Figure 6:**
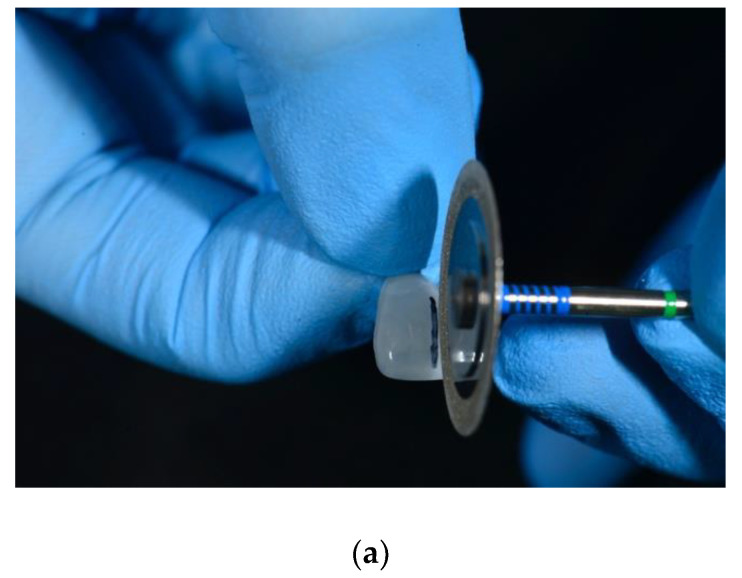

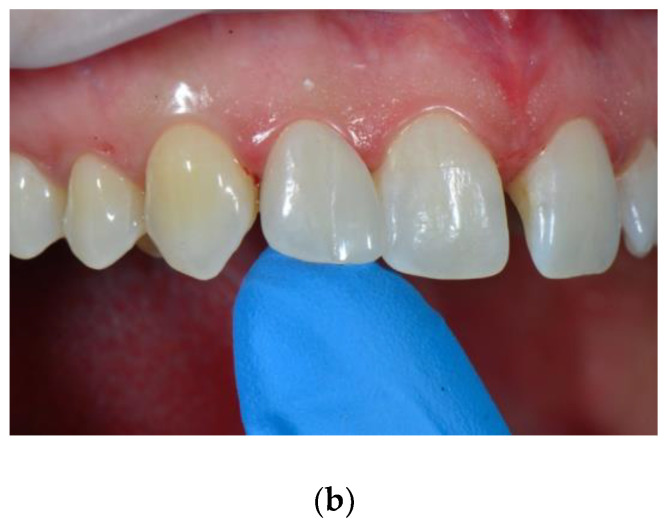
(**a**) A diamond separating disc is used to cut the veneer; (**b**) the sectional veneer clicks into place.

A diamond separating disc (ZR943, Komet, Lemgo, Germany)was used to cut the veneer along the marker line and produce a sectional veneer that exactly fits the space to be closed (Figure 6a).

After trying the sectional veneer in its eventual position to confirm the size and shape (Figure 6b), a retraction cord was packed around the tooth (Ultrapack size 00, Ultradent, South Jordan, UT, USA) to prevent contamination from the crevicular fluid and to produce the gentle displacement of the soft tissue. The cord was positioned tooth by tooth only when the veneer was ready to be delivered and was removed as soon as isolation was no longer necessary. A thin retraction cord was used since the try-in showed how the emergence profile of the sectional veneer already produced effective soft tissue compression with no residual black triangle and no need for extra soft tissue displacement. Another reason for using a thin retraction cord was the thin scalloped gingival phenotype and the risk of stressing the delicate soft tissue.

The Edelweiss Veneer requires no sandblasting, no acid etching and no silane application for bonding. However, the manufacturer recommends internal conditioning with a dedicated resin primer (Veneer Bond, Edelweiss Dentistry, Wolfurt, Austria) to promote chemical adhesion and increase bond strength between the highly inorganic composite laminate and the luting composite.

**Figure 7:**
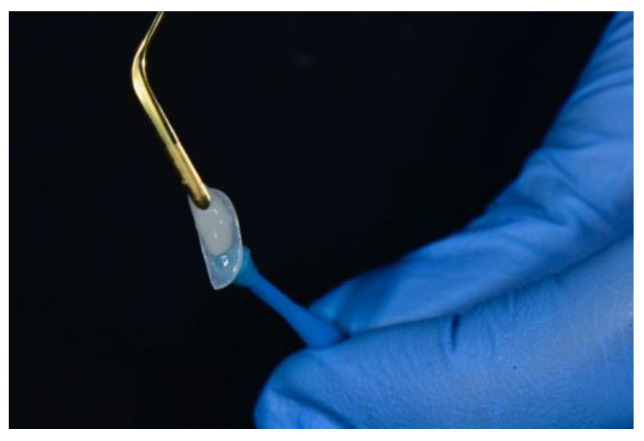
Sectional veneer loaded with enamel shade.

After Veneer Bond application, the prefabricated Veneers are ready for bonding using a complementary nanohybrid composite resin available in several dentin and enamel shades (Edelweiss NH, Edelweiss Dentistry, Wolfurt, Austria). Small- and medium-sized sectional veneers (i.e., diastema width <2 mm) are usually bonded with an enamel shade only. Larger sectional veneers are usually bonded with the combination of a dentin shade in the gingival area and an enamel shade in the incisal area in order to produce gingival–incisal color gradation and an increased background masking effect. For the patient presented in this paper, all the sectional veneers were bonded with an enamel shade only (Figure 7).

**Figure 8:**
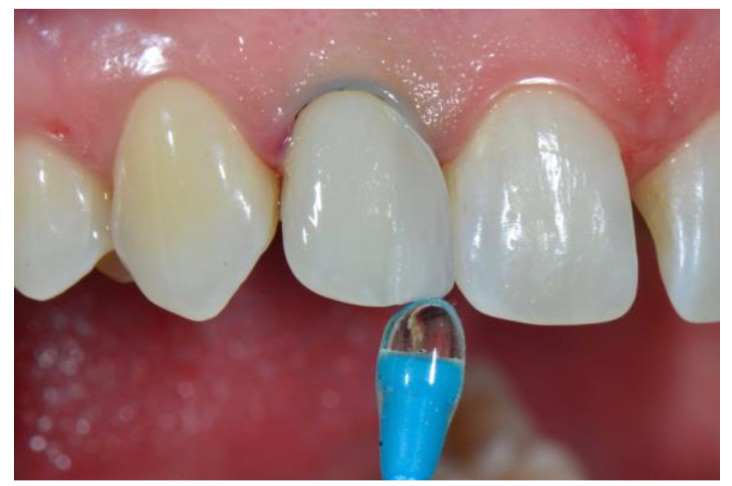
Prefabricated veneer seated in position until connection with the adjacent tooth.

**Figure 9:**
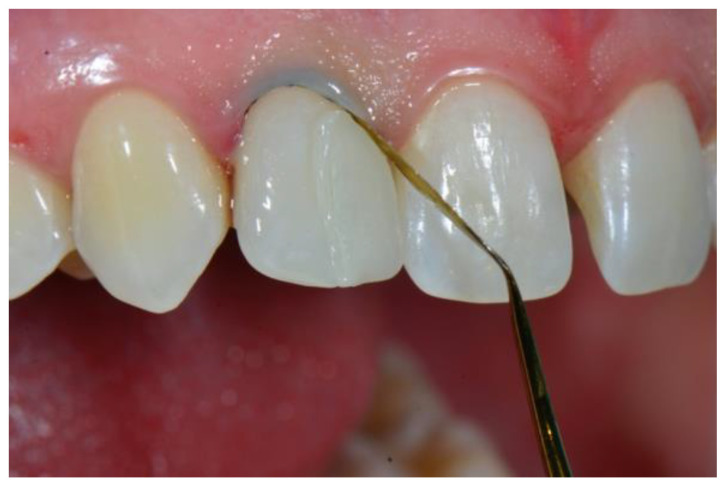
The extra composite is carefully sculpted with an ultrathin spatula.

Once the sectional veneer was loaded with the selected composite shade and was therefore ready for bonding, the tooth was etched with 35% orthophosphoric acid followed by the application of a single-step adhesive, according to the manufacturer instructions (Peak Universal, Ultradent, South Jordan, UT, USA). Then, the first sectional veneer was seated in position and gently pressed until it made contact with the adjacent tooth (Figure 8). A thin spatula (IPC, American Eagle Instruments, Missoula, MT, USA) was used to sculpt the extra composite and achieve optimal adaptation between the veneer and the tooth (Figure 9). Finally, the veneer was light cured for 20 s from the lingual direction plus 20 s from the buccal direction using a high-power curing light (Demi Plus, Kerr Corporation, Brea, CA, USA).

**Figure 10:**
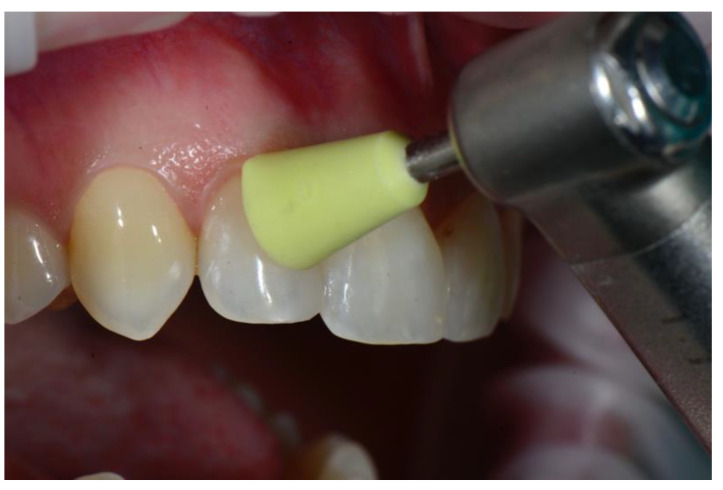
Finishing the margins.

**Figure 11:**
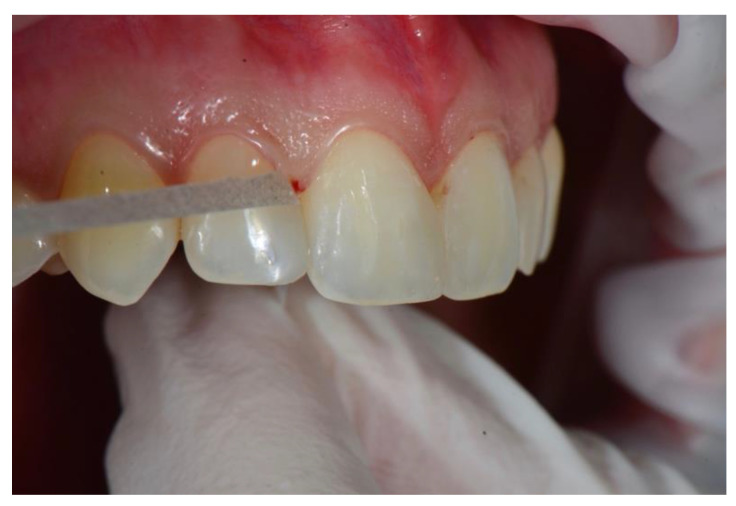
Finishing the interproximal spaces.

Once curing was completed, the margins of the sectional veneer were finished and polished with conventional composite instruments. The tested prefabricated veneers do not require special equipment and can be effectively polished with most of the commercially available polishing systems for nanohybrid composite resin. In the case presented in this report, a 50-micron needle point diamond bur (Brasseler DET3F, Brasseler, Savannah, GA, USA) was used to smooth the extra composite, followed by a silicone cup (Identoflex Composite Polisher, Ravelli, Milano, Italy) to remove the residual scratches (Figure 10) and a paper finishing strip (Sof-Lex, ESPE 3M) to polish the interproximal area (Figure 11). Finishing and polishing is limited to the margins of the sectional veneer and no instrumentation is required on the buccal and lingual surfaces, since prefabricated veneers are pre-shaped and pre-polished to the ideal anatomic form, with accurate superficial anatomy and a high-gloss, laser-sintered surface.

**Figure 12:**
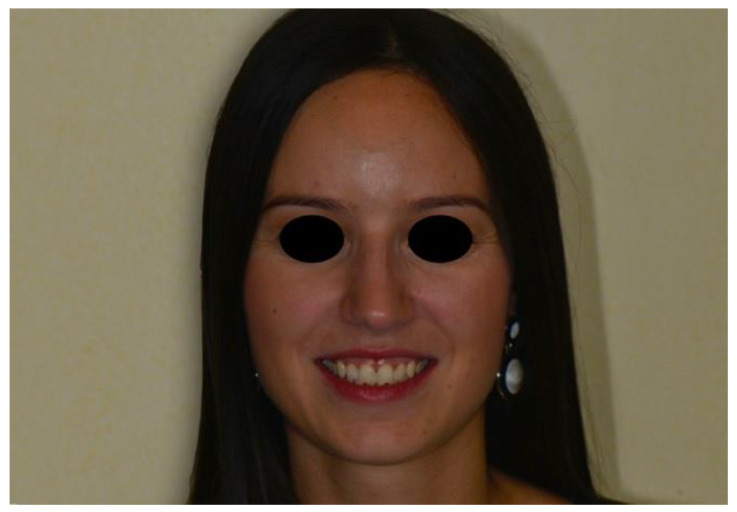
Post-operative full-face view.

**Figure 13:**
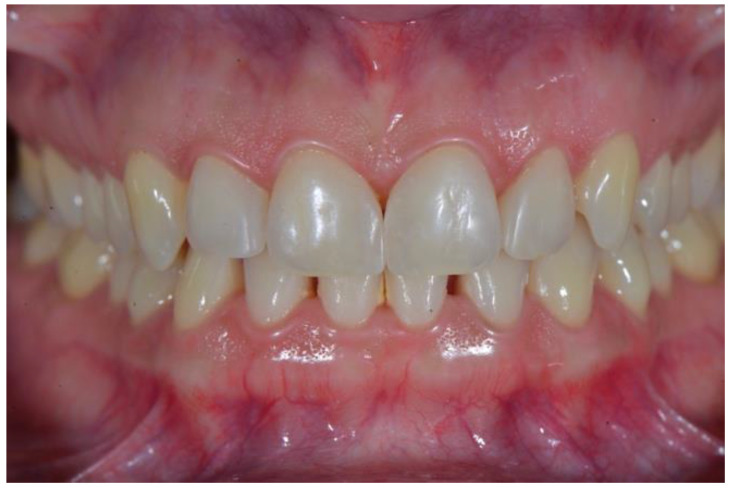
Post-operative retracted view.

Once all the veneers were bonded in position, the patient was dismissed and rescheduled for a post-operative evaluation two weeks later. At the recall appointment, a functional evaluation (absence of fractures, marginal adaptation), a biological evaluation (soft tissue response, post-operative sensitivity) and an esthetic evaluation (gloss, color matching) were completed and were fully satisfactory (Figure 12 and Figure 13). As a final recommendation, oral hygiene instructions for the interproximal dental spaces were reviewed and the patient was rescheduled for a regular 6-month follow-up appointment.

The highly translucent and glossy surface of the Veneer produced optimal esthetics with invisible transition between the veneer and the tooth. The lack of tooth preparation and the single-appointment procedure with no impressions, no temporaries and no try-ons minimized the stress for the soft tissues and greatly contributed to the reduced post-operative discomfort and excellent acceptance by the patient.

## 3. Discussion

This paper presents an alternative technique for minimally invasive diastema restoration with sectional veneers. The sectional veneer technique was introduced as a variation on the traditional ceramic veneer technique for highly esthetic veneer restoration with no need for tooth preparation [34,35]. However, despite the combination of excellent esthetics and no sacrifice of the tooth structure, ceramic sectional veneers have failed to enter the mainstream of general dentistry because of the technically demanding clinical procedure and the delicate laboratory fabrication process. Another limitation to the popularity of the ceramic sectional veneer technique is the high risk of accidental breakage and post-bonding crack formation on the knife-edged margins due to the reduced ceramic thickness and the unfavorable ceramic/composite thickness ratio [36,37,38].

In the case presented in this paper, the sectional veneer technique was reinterpreted using a prefabricated composite veneer. The prefabricated composite veneer technique was originally launched in the late 1970s (Mastique Veneer System, Caulk Dentsply) but with limited success because the large glass filler technology available at the time did not provide adequate esthetics and durability [39,40,41]. However, the concept of a prefabricated veneer was rejuvenated about 40 years later thanks to the introduction of contemporary composite resin with improved mechanical and esthetic properties and, today, several products of this category are available on the market for single-appointment veneer restoration (Edelweiss Veneer by Edelweiss Dentistry, Componeer by Coltene, Visio.Lign Veneer by Bredent) [42]. The Edelweiss Veneer used for the patient in this report was the first of this “new generation” of prefabricated veneers introduced on the market in 2011. Edelweiss Veneers feature an advanced fabrication technology with heat/pressure composite curing and laser sintering and were chosen to produce the sectional veneers for the patient in this report due to their excellent performance and resistance to fatigue in laboratory testing [43]. For the purpose of diastema restoration, a prefabricated composite sectional veneer provides the same benefits of a conventional laboratory-fabricated ceramic sectional veneer (a highly esthetic restoration with no need for tooth preparation) but without many of its intrinsic limitations. First of all, the veneer’s composite resin mechanical properties allow the dentist to fabricate a less delicate restoration with a reduced risk of post-bonding crack formation on the knife-edged margins because the composite low flexural modulus yields a better absorption of the forces generated by thermal and polymerization stress [44,45,46]. Another benefit of using a prefabricated composite veneer for sectional veneer fabrication and diastema restoration is the less technically demanding and time-consuming clinical procedure, involving a single appointment with a reduced number of clinical steps. A prefabricated composite veneer requires no impressions, no temporaries and no try-ons, and can be bonded like a direct composite restoration with no need for sandblasting, acid etching, post-etching cleaning or silane application. Furthermore, finishing and polishing the margins of a prefabricated composite sectional veneer are less technically demanding and time consuming than finishing and polishing the margins of a ceramic sectional veneer, since the procedure is not influenced by many variables (type of ceramic, type of polisher, polishing speed/pressure) [47,48] and can be accomplished using standard polishing systems for nanohybrid composite resin with no risk of adversely affecting the esthetic and mechanical properties and of initiating internal crack propagation [49,50]. On the other hand, a ceramic sectional veneer is expected to provide better longevity than a composite sectional veneer thanks to ceramic’s superior mechanical properties and color stability. In clinical studies, ceramic veneers show excellent clinical performance [51,52,53] and, in a recent systematic review and meta-analysis, the estimated overall cumulative survival rate of glass–ceramic and feldspathic porcelain veneers was 89% in a median follow-up period of 9 years [54]. However, at the moment, there is no clinical study reporting on the longevity of ceramic sectional veneers and it is possible that their survival rate would not be as good as conventional ceramic veneers because laboratory studies show high stress concentrations at the bonding interface of ceramic sectional veneers [55]. In a three-dimensional finite element analysis, ceramic sectional veneer diastema restoration shows a significantly higher stress concentration than direct composite diastema restoration and the larger the size of the diastema, the higher the von Mises stress value. Stress distribution is concentrated along the margins of the sectional veneer, leading to possible debonding or accelerated marginal degradation [56,57].

Possible clinical alternatives to the sectional veneer approach would have been orthodontic space closure with conventional [58] or miniscrew-assisted [59] orthodontic therapy. However, these choices would have been more time-consuming and would have caused aesthetic discomfort due to the presence of the appliance. Additionally, during both vestibular [60] and lingual [61] orthodontic therapies, dental hygiene procedures are more complex and oral microbiology could change.

Clinical studies are available for diastema restoration made with direct composites and show acceptable clinical performance and longevity: Frease et al. scored an 84.6% survival rate at 5 years with a clinical quality rated from good to excellent for most of the diastema restorations examined [62], Peumans et al. reported an 89% survival rate at 5 years [63], Gresnigt et al. found a survival rate of 87.5% after a mean observation period of 41.3 months [44]. The prefabricated composite veneer used in this case report is expected to provide even better longevity than conventional direct composite diastema restoration because the incorporation of heat and pressure in the curing process increases the filler density and monomer conversion rate with a positive impact on physical properties and color stability [64,65,66]. It is as yet unknown to what extent the technology involved in the fabrication of Edelweiss prefabricated veneers effects clinical performance, since this technique was recently introduced on the market and clinical studies are not yet available. It is possible that a diastema restoration made with a traditional ceramic sectional veneer still outperforms a diastema restoration made with an Edelweiss prefabricated composite sectional veneer because of its higher mechanical strength and better resistance to aging. However, for many dentists and many patients, the gap in longevity could be compensated by the benefits of a restoration that involves a less technically demanding clinical procedure, a less time-consuming technique, possible intraoral repair and a more affordable cost for the patient.

## 4. Conclusions

The concept of successful restoration in contemporary dentistry embraces not only the traditional criteria of minimal biological cost, good longevity and successful esthetic integration, but also other factors, including the uncomplicated technique, possible intraoral repair, reduced soft tissue trauma and affordable financial cost. It is up to the dentist to balance all these factors and select the best treatment for the patient. However, the wider the choice of treatment options, the easier for the dentist to select the best treatment based on the clinical needs and for the patient to choose the best treatment based on his esthetic expectations and financial possibilities. The technique presented in this paper implements the traditional choice of either a direct composite or indirect ceramic and introduces an additional treatment option to produce a noninvasive diastema restoration in a single appointment, with a reduced number of clinical steps.
